# Modelling of Epithelial Growth, Fission and Lumen Formation During Embryonic Thyroid Development: A Combination of Computational and Experimental Approaches

**DOI:** 10.3389/fendo.2021.655862

**Published:** 2021-06-07

**Authors:** Leolo Gonay, Catherine Spourquet, Matthieu Baudoin, Ludovic Lepers, Pascale Lemoine, Alexander G. Fletcher, Emmanuel Hanert, Christophe E. Pierreux

**Affiliations:** ^1^ Earth and Life Institute, UCLouvain, Louvain-La-Neuve, Belgium; ^2^ de Duve Institute, UCLouvain, Woluwé-Saint-Lambert, Belgium; ^3^ School of Mathematics and Statistics, University of Sheffield, Sheffield, United Kingdom

**Keywords:** modelling, morphogenesis, thyroid, epithelial, vertex model

## Abstract

Organogenesis is the phase of embryonic development leading to the formation of fully functional organs. In the case of the thyroid, organogenesis starts from the endoderm and generates a multitude of closely packed independent spherical follicular units surrounded by a dense network of capillaries. Follicular organisation is unique and essential for thyroid function, *i.e.* thyroid hormone production. Previous *in vivo* studies showed that, besides their nutritive function, endothelial cells play a central role during thyroid gland morphogenesis. However, the precise mechanisms and biological parameters controlling the transformation of the multi-layered thyroid epithelial primordium into a multitude of single-layered follicles are mostly unknown. Animal studies used to improve understanding of organogenesis are costly and time-consuming, with recognised limitations. Here, we developed and used a 2-D vertex model of thyroid growth, angiogenesis and folliculogenesis, within the open-source Chaste framework. Our *in silico* model, based on *in vivo* images, correctly simulates the differential growth and proliferation of central and peripheral epithelial cells, as well as the morphogen-driven migration of endothelial cells, consistently with our experimental data. Our simulations further showed that reduced epithelial cell adhesion was critical to allow endothelial invasion and fission of the multi-layered epithelial mass. Finally, our model also allowed epithelial cell polarisation and follicular lumen formation by endothelial cell abundance and proximity. Our study illustrates how constant discussion between theoretical and experimental approaches can help us to better understand the roles of cellular movement, adhesion and polarisation during thyroid embryonic development. We anticipate that the use of *in silico* models like the one we describe can push forward the fields of developmental biology and regenerative medicine.

## Introduction

The thyroid is a highly vascularised endocrine gland responsible for the synthesis of triiodothyronine (T3), thyroxine (T4) and calcitonin. These hormones are crucial in human growth and development, particularly in young children, but also in regulation of basal metabolism (1–3). In humans, as in most mammals, the adult thyroid consists of two lobes (left and right) positioned anteriorly in the lower-neck region, close to the trachea. The two lobes are connected by a thin layer of median tissue called the isthmus. The functional unit of the thyroid is called the follicle. It is a spherical structure composed of a single layer of epithelial follicular cells, the thyrocytes, resting on a continuous basement membrane and surrounding a central cavity. This cavity, or lumen, contains the colloid, a homogeneous non-crystalline substance consisting of large iodinated molecules of thyroglobulin (Tg) that constitute the thyroid hormone reservoir. The basement membrane that surrounds the follicular cells is a thin, non-cellular matrix that separates the epithelial monolayer from the underlying connective tissue. It is mainly composed of collagens, fulfilling a structural role, and laminins, forming cross-shaped and multi-adhesive structures ensuring adhesion with the epithelial surface. Beyond the basement membrane, the connective tissue contains fibroblasts, blood vessels and various secreted proteins. Together with the basement membrane, these proteins form the extra-cellular matrix (ECM). Thyrocytes, the most abundant cell type in the thyroid gland, are differentiated, polarised endocrine cells that produce the thyroid hormones T3 and T4. Each thyrocyte possesses an apical surface facing the colloid, a basal surface facing the basement membrane and the connective tissue, and a lateral pole interacting with other thyrocytes in the follicle (1–3). These cells form a tight barrier between the colloidal space of the follicle and the extra-follicular connective tissue. The cohesion of the thyrocyte monolayer in ensured by different junctional complexes such as the adherens junctions (or *zonula adherens*), mainly formed by E-cadherins. A second glandular cell type, the calcitonin-producing C-cell, is found in the thyroid. These cells are found interspersed between the follicles with which they are closely associated (1–3).

Progenitors of the thyrocyte and C-cell lineage originate from different endodermal structures. Thyrocyte progenitors bud from a midline region in the pharyngeal floor while C-cells arise from the fourth lateral pharyngeal pouches (4, 5). In most mammals, five stages can be distinguished during thyroid morphogenesis: specification, budding, migration, bilobation and folliculogenesis (5). Note that the morphogenetic stages of human and mouse thyroid development are roughly similar, but with different timing. Around embryonic day (E) 8.5 in mice (around the 4^th^ week in human), the thyroid placode is formed close to the base of the tongue (specification). At E9.5, this placode converts into a bud or thyroid primordium (budding) and at E10.5, thyroid bud migrates caudally and detaches from the pharyngeal floor. Around E12.5-E13.5 (7^th^ – 8^th^ week in human), the thyroid primordium bifurcates and migrates laterally on both sides of the trachea and fuse with the C-cell precursors (grouped in a structure called the ultimobranchial body). On each side of the trachea, the fusion leads to the formation of a thyroid lobe (bilobation) (5). The lobes increase in size by rapid proliferation and thyroid epithelial cells progressively acquire polarity and organise to form the follicles.

Formation of the thyroid lobes coincides with the development of a rich network of endothelial cells (ECs) (6). ECs are generally recruited by epithelial organs *via* secretion of growth factors such as VEGFA (Vascular Endothelial Growth Factor) and are induced to form new blood vessels in a process called angiogenesis. The combined process of production, diffusion and degradation of growth factors creates a gradient across the width of the epithelial tissue (7, 8). This gradient causes the activation of some endothelial cells (which will differentiate and become tip cells) in blood vessels localised in the vicinity of the epithelial tissue. Tip cells thus change shape and migrate in the direction of the gradient, closely followed by other dividing endothelial cells, called stalk cells, from the blood vessel (7, 8). Our laboratory demonstrated that the recruitment and expansion of ECs during thyroid development depends on the expression of VEGFA by epithelial cells (9). Indeed, genetic inactivation of VEGFA in the thyroid causes a dramatic decrease in thyroid endothelial density at E17.5. Moreover, this decrease in thyroid endothelial density negatively impacts on the organisation of epithelial cells into pre-follicular structures at E15.5 and eventually into large polarised epithelial monolayers (follicles) around birth (9). We further showed that the assembly of the basement membrane, partly controlled by endothelial cell invasion in the thyroid, was crucial for epithelial cell orientation, acquisition of polarity and *in fine* follicle formation (10).

Despite numerous studies conducted in the field of developmental biology and organogenesis, the precise mechanisms and biological parameters controlling organ formation are still unclear. Moreover, hypothesis-driven experimentation using animal models is always time-consuming, frequently technically challenging and sometimes not feasible at all. There is thus a growing need to employ complementary approaches to improve our understanding of tissue morphogenesis. One such approach, computational modelling, offers a way of integrating static observations within a dynamic framework, and has proven a powerful tool aiming to help unlock, among others, complex biological systems (11, 12). *In silico* models can be of great interest when it comes to testing and confronting various hypotheses regarding the complex processes at stake, without the limitations of *in vitro* or *in vivo* experiments. To the best of our knowledge, such a mathematical model has not been developed for thyroid morphogenesis. In this context, we sought to develop a 2D vertex-based model of murine thyroid growth, fission, angiogenesis and folliculogenesis. We expect that this model, developed step-by-step at the edge of experimental and computational biology, could be further used to investigate the mechanisms and biological parameters controlling the reorganisation of the multi-layered thyroid epithelial primordium into a multitude of single-layered follicles.

## Material and Methods

### Experimentations and Measurements

#### Animals

All mice were of the CD1 strain. Mice were raised and treated according to the NIH Guide for Care and Use of Laboratory Animals, and experiments were approved by the University Animal Welfare Committee, Université Catholique de Louvain (2016/UCL/MD/005 and 2020/UCL/MD/011).

#### EdU Injection

Mice were injected with a solution of EdU (Invitrogen) 25mg/ml at embryonic stages E13.5, E15.5 and E17.5. The solution was heated up to 70°C for 1 min to completely dissolve the compound. When at room temperature, the solution was intraperitoneally injected to the mice (100 μg EdU/g of mouse). Mice are sacrificed after 30 min by cervical dislocation. Mouse embryos are microdissected as described in (13).

#### Embryos Fixation, Section, and Immunolabelling

Mouse embryos were fixed overnight at 4°C by paraformaldehyde 4% before paraffin embedding. Embryos sections were performed every 6 μm, collected on Superfrost+ slides and kept overnight at 37°C. For immunolabelling, sections were successively submerged in xylene (3 X 5 min), isopropyl alcohol (30 sec) and then re-hydrated in ethanol (90%, 70%, 30%, 3 min each) and then water. Sections were treated to allow target accessibility (un-masking) by citrate buffer 0.01 M at pH 6 while being heated up. Sections were then permeabilised by a solution PBS/Triton X-100 0.3% on an agitator plate. Finally, a blocking solution PBS -/Triton X-100 0.3%/BSA 10%/milk 3% was applied for 60 min. Antibodies, dilutions and immunolabeling protocol are described in (9, 10, 14) (See **Supplementary Table 1**). EdU was revealed using the Click-iT™ Plus EdU Alexa Fluor™ 488 Imaging Kit (Invitrogen). Sections were scanned with a Panoramic 250 Digital Slide Scanner (3DHistech).

For quantification of EdU-positive and ezrin-positive cells, ezrin-labelled structures and endomucin-positive area, we used the image analysis platform HALO®^1^ with its appropriate in-built algorithms; Indica Labs CytoNuclear FL for cytoplasmic and nuclear immunofluorescence and Indica Labs Area Quantification for endothelial density quantification. Quantification of the number of epithelial cells per lumen was performed by manual counting on HALO. These data were used in our calibration and validation processes.

### Computational and Statistical Analysis

Statistical analysis and data pre-processing for the generation of our initial conditions (IC) were performed using scripts developed in MATLAB^®^ ^2^. Data are presented as mean ± s.d. Our computational model was written in C++ using the open-source library Chaste^3^. Simulation results were visualised in ParaView^4^.

### Vertex Model

Vertex models (VMs) are a widely-used approach for describing the dynamics and remodelling of epithelial tissues (15). Here, we used a 2D VM to simulate the evolution of a 2D cell sheet, representing a cross-section in the developing thyroid. We decided to use 2D modelling as 3D models demand more computational resources and 3D images are more complex to obtain and quantify, while 2D models appeared sufficient to study the processes driving follicles formation. In VMs, cell is approximated by a polygon whose vertices move in response to forces due to growth, interfacial tensions or hydrostatic pressure. Our VM, detailed below, is based on (16).

#### Equation of Motion

In our VM, edges represent the plasma membrane, while vertices correspond to adherens junctions where three or more cells meet. Each cell’s shape and location change over time due to the movement of its vertices. Assuming overdamped dynamics and supposing that adhesion is strong enough that random fluctuations in vertex position can be neglected (17), the evolution over time of the position ${\vec{r}}_{k}$ of a vertex *k* is given by

(1) $$\frac{d{\vec{r}}_{k}}{dt}=\;{\vec{F}}_{k}(t)\;,$$

where ${\vec{F}}_{k}(t)$ denotes the net force on vertex *k* at time *k*. In our VM, forces acting on each vertex are derived from the minimisation of the total energy stored in the whole tissue (16). In other words, this approach evaluates the energy stored both inside the cells and along their membranes. The total energy of the cellular population is a function of the location of each vertex and is closely related to the work needed to deform the cellular network. It is defined as:

(2) $$E=\sum_{Cells\;\alpha }\frac{K}{2}{\left(A_{\alpha }-A_{0}\right)}^{2}+\sum_{Edges\;j}\Lambda_{j}\cdot L_{j}+\sum_{Cells\;\alpha }\frac{\Gamma }{2}P_{\alpha }^{2},$$

where *K* is an elasticity parameter, *A_a_* and *P_a_* denote respectively the area and perimeter of cell *α*, *A*_0_ is a cell’s ‘target area’, Λ*_j_* is a line tension parameter associated with edge *j*, *L_j_* is the distance between two vertices and Γ is a contractility parameter.

The force acting on vertex *k* is then derived from the energy gradient:

$${\vec{F}}_{k}=\;-{\vec{\nabla }}_{k}E,\;foreach\;vertex\;k\;at{\vec{r}}_{k}$$

By computing the energy gradient, we obtain an explicit expression of the force acting on each vertex:

(3) $${\vec{F}}_{k}=\;\sum_{Cells\;\alpha }\left(-K_{\alpha }\left(A_{\alpha }-A_{0}\right){\vec{\nabla }}_{k}A_{\alpha }-\Gamma_{\alpha }P_{\alpha }{\vec{\nabla }}_{k}P_{\alpha }\right)-\sum_{Edges\;j\;}\Lambda_{j}{\vec{\nabla }}_{k}L_{j}$$

where, in practice, the only non-zero contributions to the first and second sums come from cells and edges sharing with vertex *k*, respectively.

#### Rearrangement, Growth and Division

Five types of operations, called junctional rearrangements, can be performed on vertices and/or edges to ensure that cells never intersect and that they are able to form and break bonds with other cells [as detailed in (18)]. First, T1 swaps (or edge rearrangements) occur when two vertices are located too close to each other. This operation ensures that any vertex located away from the boundary of the tissue is shared by exactly three cells and that any vertex located on the boundary of the tissue is shared by one or two cells. Second, T2 swaps allow a cell with three edges to be removed from the tissue (corresponding to cell death or delamination) if its area become smaller than a threshold value. Third, T3 swaps allow edges to merge, preventing vertex-edge intersections at the boundary of the tissue. Fourth, cell division is achieved by defining a dividing line (being the shortest axis through the cell’s centroid) and generating two new vertices at the intersection between this dividing line and the mother cell edges, generating two new cells of equal area (19). Fifth, the formation and resolution of multicellular rosettes, a generalisation of T1 transitions involving a larger number of cells.

#### Cell Types

In the thyroid we will consider two cell populations (epithelial and endothelial) and the follicular lumina. These three elements are distinctly described in our simulations as endothelial (tip and stalk) cells, epithelial (central and peripheral) cells, and lumen cells (see *Results* and *Discussion*). Each cell in the population is labelled accordingly and these cell types are associated with specific characteristics such as their target area, cell cycle definition or adhesion parameters. The implementation of various cell types allowed to simulate differential adhesion by using different values for the energy parameters in eq. (2), depending on the cell types sharing edge *j* [in line with previous VMs of differential adhesion (20)].

The resulting system is solved using a forward Euler discretisation in time with a time step small enough to ensure numerical stability and accuracy (18).

#### Cell Polarity

As the polarity of cells *in vivo* change with thyroid folliculogenesis, in our model the magnitude and the direction of the polarity vector must be constantly updated in every epithelial cell as a consequence of its interaction or contact with the neighbouring endothelial and epithelial cells, with the boundary of the epithelium, and with developing lumina. In short, any epithelial cell will see its vector modulated by a certain value (depending on various parameters calibrated using our experimental data, see **Supplementary Table 2**). The magnitude and direction of the polarity vector will be affected in two main situations. On the one hand, the polarity vector $\vec{p}$ of an epithelial cell *α* will point away from the centre of its neighbouring endothelial cell *β* or with the epithelial mass periphery:

(4) $$\{\begin{array}{c} p_{x}(t)=\;p_{x}(t-\Delta t)+\;\rho \frac{d_{x}}{d(\alpha ,\;\beta )}\Delta t\\ p_{y}(t)=\;p_{y}(t-\Delta t)+\;\rho \frac{d_{y}}{d(\alpha ,\;\beta )}\Delta t \end{array}$$

with *ρ* being de polarisation rate, *d*(*α*, *β*) the distance between the centre of the epithelial cell *α* and the neighbouring cell *β* and *d_x_* and *d_y_* the components of *d*(*α*, *β*) respectively along the *x* and *y* axis. On the other hand, the polarity vector of a cell facing a strongly polarised cell or facing a well-developed lumen will be oriented in the opposite direction to the vector of the polarised cell (as defined in eq. (5)) or to the lumen (with this time a negative value for *ρ* in eq. (4)). The impact of neighbouring epithelial cells on the polarity vector of any epithelial cell is defined as:

(5) $$\vec{p}(t)=\;\vec{p}(t-\;\Delta t)\mp \epsilon \cos(\theta )\Delta t$$

with *ϵ* being the polarisation rate related to epithelial cells and *θ* the angle between *α* and any neighbouring epithelial cell.

All computational scripts and codes are available upon request.

## Results

### Generation of Image-Based Initial Conditions for the VM

**Figure 1:**
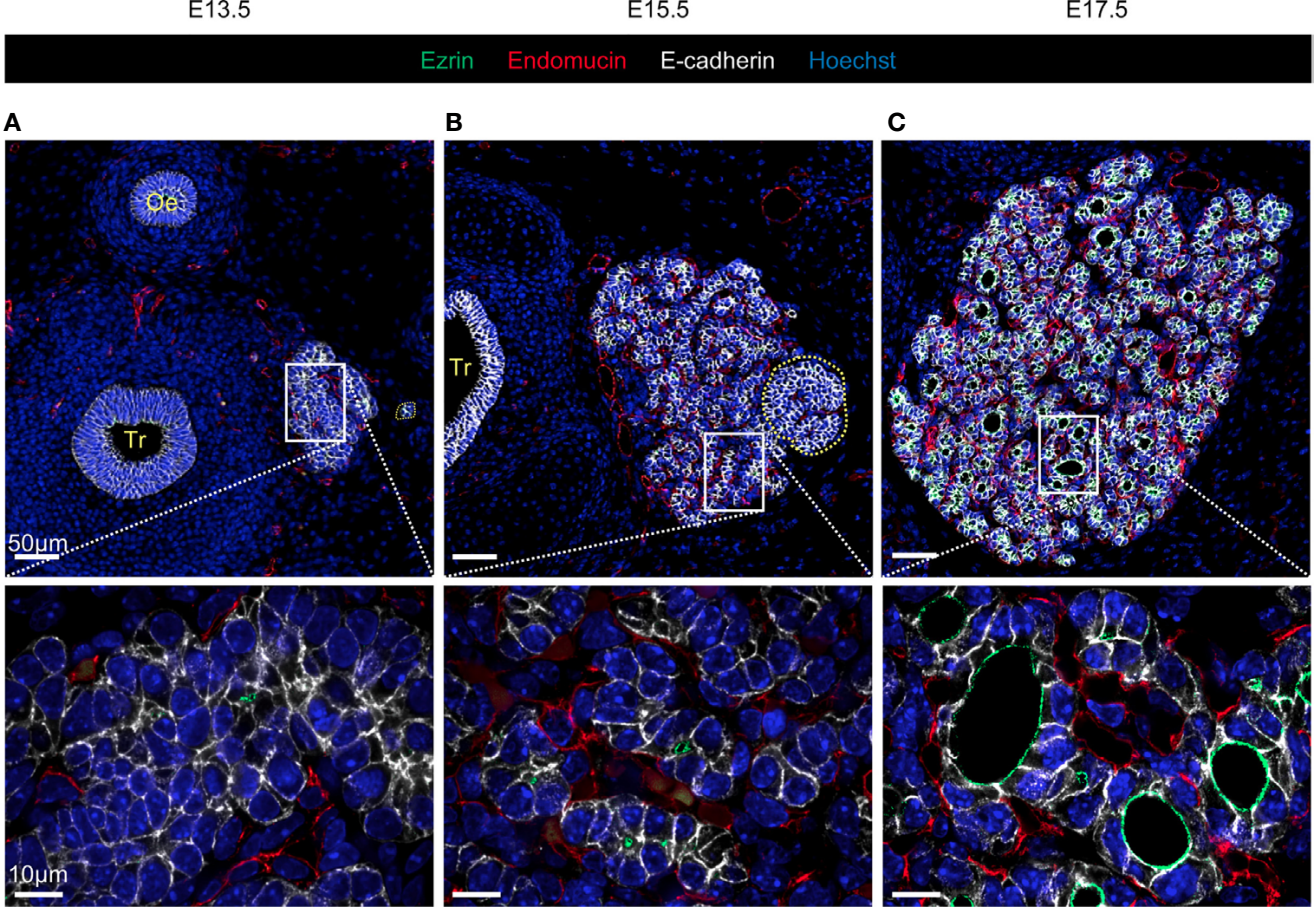
Visualisation of the developing thyroid at three different embryonic stages. Immunolabelling of epithelial cells in white (E-cadherin), endothelial cells in red (Endomucin), and of the apical pole of epithelial cells in green (Ezrin). Nuclei are stained in blue (Hoechst). Tr, trachea; Oe, oesophagus. **(A)** At E13.5, non-polarised epithelial cells are organised in a multi-layered mass (here, the left thyroid lobe is shown). Sections through endothelial structures are visualised around the thyroid lobe. Magnification revealed that only a few cells present traces of Ezrin labelling, but no intercellular lumen. **(B)** At E15.5, the thyroid lobe has greatly increased in size and is progressively losing its multi-layered aspect. Dotted yellow line mark the parathyroid gland. Magnification revealed that the thyroid is now fragmented in cords or smaller masses and that epithelial cells are in contact with endothelial cells (red). Ezrin+ structures are now visible and represent the first small interepithelial lumina. **(C)** At E17.5, the thyroid has reached an almost mature configuration, with epithelial cells organised in monolayers around a central lumen (follicular lumen in green) and surrounded by a dense network of endothelial cells (red).

Cells in the vast majority of VMs are initialised with a honeycomb, *i.e.* perfectly hexagonal, shape (21). Using such an initial configuration has the advantage of being easily implemented and reproduced. However, while these allow for a somewhat acceptable simple representation of epithelial sheet configuration, the biological reality is clearly more complex, as exemplified by the developing thyroid (**Figure 1** and **Supplementary Figure 1**). The three stages presented (E13.5, E15.5 and E17.5) illustrate the biological complexity of thyroid organogenesis with (i) epithelial cell growth (epithelial cells are visualised in white), (ii) transformation of a mass of cells into polarised spherical structures (apical pole is shown in green), coupled with (iii) massive endothelial angiogenesis (red in **Figure 1**), and (iv) basement membrane deposition (red in **Supplementary Figure 1**). Hence, we exploited the availability of high-quality experimental images and image analysis software to provide an initial cell packing that more faithfully represented the tissue architecture *in vivo*.

**Figure 2:**
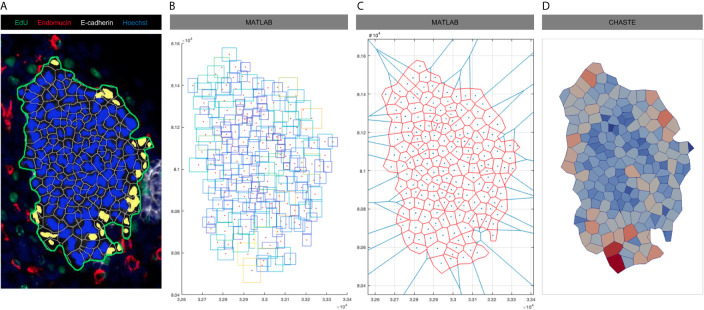
Process of initial condition generation from microscopic imagery. **(A)** E13.5 thyroid section labelled for E-cadherin (white), Endomucin (red), EdU (green) and nuclei (blue). Epithelial cell nuclei were coloured in blue and endothelial cell nuclei in yellow using HALO. The area of analysis is delineated by a green line **(B)** Rectangles enclosing epithelial and endothelial cells identified in **(A)** were recreated in MATLAB with a red dot localized in the centre of each of these rectangles. Contours of rectangle are coloured according to their area (increasing from blue to yellow). **(C)** Voronoi diagram was generated in MATLAB using cell centres from **(B)** as seeds. Original tesselation in blue, final tesselation using the manual annotation of the area of analysis (A, in green) as boundary in red. **(D)** Visualisation of the final cell population in Chaste that will be further used as initial condition in our simulations. Cell are coloured according to their area (increasing from blue to red).

We therefore developed a method to generate more biologically-realistic IC from microscopic images of the thyroid (**Figure 2**). This method enables the transformation of any images processed in the image analysis platform HALO into a numerical structure that can be further used in Chaste as initial condition (IC) for our VM. Images are first analysed in HALO using its built-in algorithm to detect epithelial cells inside a specific area of analysis (**Figure 2A**). The position of a rectangle enclosing each previously detected epithelial cell is then exported and can be used in any other programming environment (**Figure 2B**). The centre of each of these rectangles is then used as Voronoi seeds to recreate the cells detected *in vivo* (**Figure 2C**) using the area of analysis in **Figure 2A** (in green) as boundaries. A Voronoi tesselation generates a partition of a plane into regions (cells) that consist of all points that are closer to a given object (seed) than to any other object on this plane. The annotation in **Figure 2A** is used to close the initial tesselation (which previously showed infinite cells on the boundaries). This closed Voronoi tessellation eventually generates a digitalised cell distribution than can be used as an initial cell packing (including vertex positions and topology) in our VM simulations (**Figure 2D**).

### Quantification of Cell Proliferation

**Figure 3:**
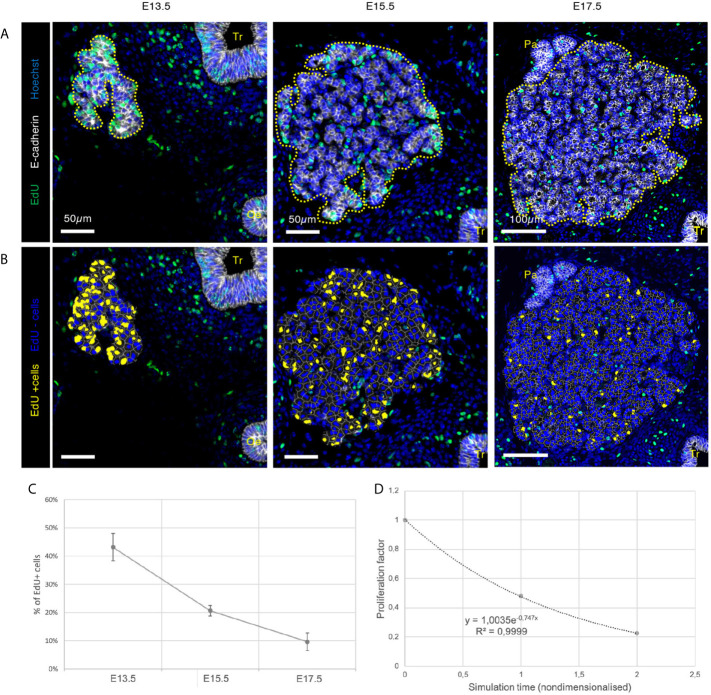
Visualisation and quantification of proliferating cells in developing thyroid at the three different embryonic stages. **(A)** Immunolabelling of epithelial cells in white (E-cadherin) and proliferating cells in green (EdU). Nuclei are stained in blue (Hoechst). Tr, trachea; Oe, oesophagus; Pa, parathyroid. **(B)** EdU+ cells were coloured using HALO in yellow and EdU- cells in blue. **(C)** Percentage of EdU+ cells in the developing thyroid at the three embryonic stages (n = 3). **(D)** Proliferation factor of epithelial cells in the embryonic thyroid. Both axes are nondimensionalised by (y) dividing the percentage of EdU+ cells by the highest value (here at E13.5), and (x) replacing the embryonic days by the time elapsed between two observations (here 48h/unit).

A large majority of VMs that includes cell division is usually based on the simplified assumption that cell division occurs stochastically, with a characteristic mean and variance (18). Here, we experimentally quantified thyroid epithelial cell proliferation using EdU labelling on mouse embryo sections at E13.5, E15.5 and E17.5 (**Figure 3A**) to localise proliferating cells and infer epithelial cell cycle duration. We used computational tools (*i.e.* HALO built-in algorithms) to detect labelled cells, to precisely map proliferating cells in the sections (**Figures 3B**) and verify the existence of two “populations” of dividing cells. Indeed, recent work has suggested the existence of differential proliferation patterns between central and peripheral cells in the developing thyroid (22) and pancreas (23).

Globally, using the same incorporation time, we observed a notable decrease in the fraction of EdU^+^ cells with thyroid development (**Figures 3A–C**). At E13.5, around 45% of the cells were labelled with EdU, indicating that these cells were in the S-phase of the cell cycle within the 30 minutes interval of the labelling period. At E15.5, this percentage dropped to around 20% and, at E17.5, to 10% of cells in S-phase. We also found by regression analysis that the proliferation rate tended to follow an exponential decrease over time that would reach values close to zero at birth (E19.5 or postnatal day 0) or shortly thereafter (**Figure 3D**). These data also confirmed the existence of two distinct cell types within the developing thyroid: central and peripheral cells. Hence, in our model, we will create two computational cell types, with specific properties. Peripheral cells will be defined as an element (a cell) with at least one of its edges (linking two vertices together) that is not shared with another cell. A central cell will be surrounded by other epithelial cells with whom it will share all of its edges.

Furthermore, we found that proliferation decreased similarly in the two populations, over the period analysed (not shown). On the contrary, the fraction of proliferating cells differed between the two populations. In fact, more than 60% of peripheral cells were proliferating at E13.5 compared to less than 40% of the central cells. At E15.5, it was almost 30% of peripheral cells that proliferated as compared to less than 10% in the centre. At E17.5, proliferation rate was slightly above 10%. Note that at E17.5, we assumed that all the cells were peripheral since at this stage prefollicular structures are formed and epithelial cells are therefore supposedly all in contact with the ECM. Altogether, these results indicate that proliferation preferentially occurred at the periphery of the thyroid mass from E13.5 onwards.

Importantly, these topological and quantitative data on cell proliferation, combined with quantification of total cell numbers, allowed us to calculate typical cell cycle lengths for the two cell populations. Using data collected at E13.5 and E15.5, we calculated a typical cell cycle duration of 11 hours and 20 minutes for peripheral cells and 31 hours and 52 minutes for central cells. These differences in cell cycle lengths, *i.e*. the time elapsed between two cell divisions, were specified to the peripheral and central cell types of our computational model in order to provide spatial and temporal precisions on thyroid epithelial growth. Thus, each epithelial cell is characterised by a phase-dependent cell cycle, which depends on the cell location. To account for the exponential decrease observed *in vivo* with developmental time (**Figures 3A–D**), the total duration of the cell cycle for both cell populations is multiplied by a generation factor. This ensures that for each new generation, cells will proliferate less and less rapidly until they differentiate and stop dividing towards the end of the simulation.

### Endothelial Recruitment and Expansion

**Figure 4:**
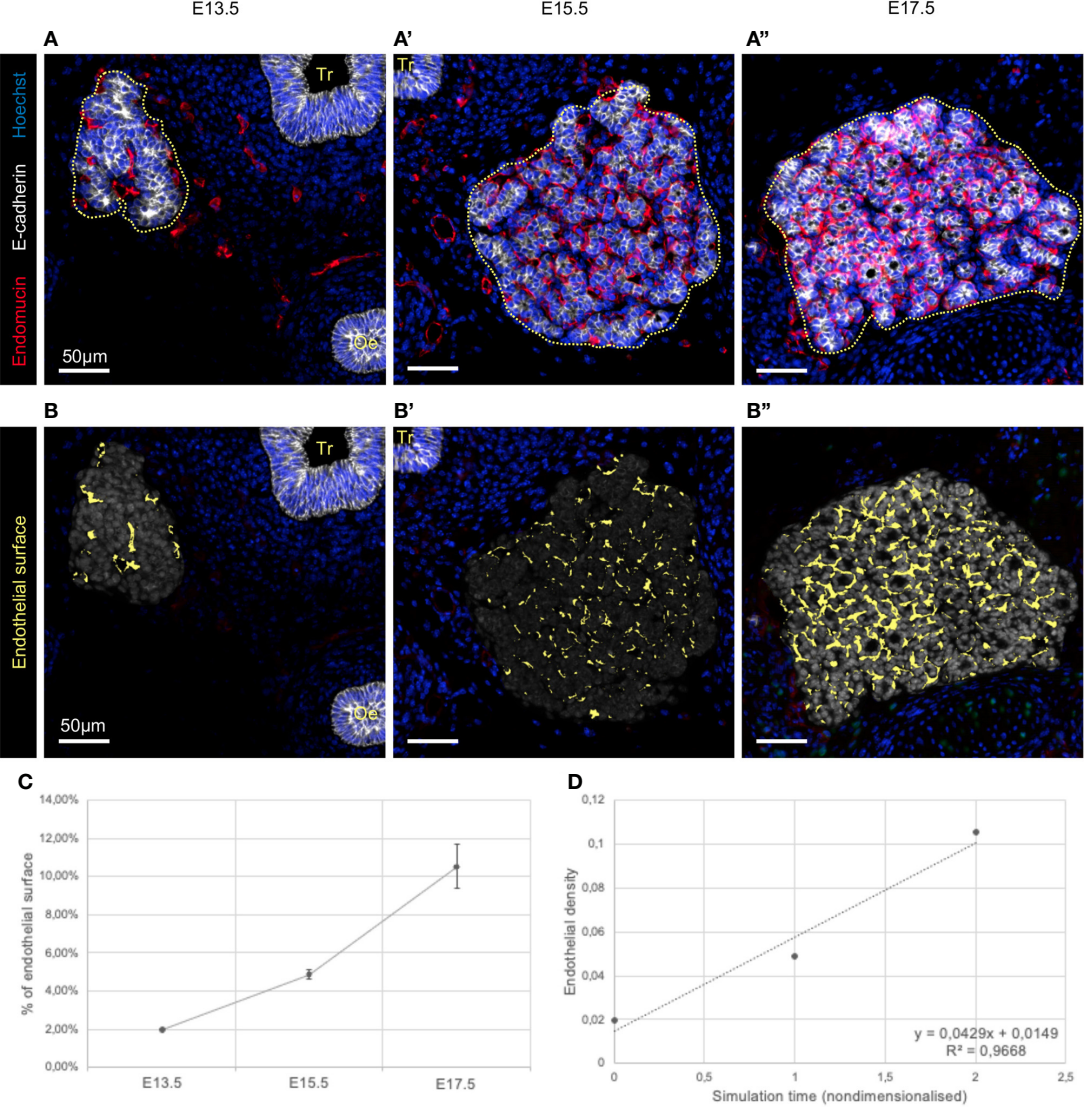
Visualisation and quantification of endothelial cells in developing thyroid at three different embryonic stages. **(A–A’’)** Immunolabelling of epithelial cells in white (E-cadherin), and endothelial cells in red (Endomucin). Nuclei are stained in blue (Hoechst). Tr, trachea; Oe, oesophagus. **(B–B’’)** Endothelial cell within the analysed region of interest (in grey) were coloured in yellow using HALO. **(C)** Percentage of endothelial cell in the region of interest (n = 3). **(D)** Endothelial density dynamics in the embryonic thyroid. Both axes are nondimensionalised by using (y) a cell area density (dimensionless), and (x) replacing the embryonic days by the time elapsed between two measures (here 48h/unit).

Along with epithelial cells, endothelial cells (ECs) needed to be integrated in our model of the endocrine thyroid gland. In fact, besides their role as constituents of nutritive pipe, ECs are known to play a perfusion-independent role in developing organs such as the thyroid (9), the pancreas (24, 25), the liver (26) and the lungs (27). We therefore imaged the developing thyroid from E13.5 to E17.5 using epithelial (E-cadherin, in white) and endothelial (Endomucin, in red) markers (**Figures 4A–A’’**).

Computational analysis of the images allowed precise mapping of endothelial cells (**Figures 4B–B’’**) and quantifications of endothelial expansion (**Figure 4C**). We observed that endothelial cells represented less than 2% of the analysed area at E13.5 (**Figure 4B**). However, endothelial cells were also found at a distance from the thyroid epithelial primordium at this stage. Subsequently, endothelial cells filled almost 5% of the space at E15.5 (**Figures 4B’, C**) and this value continued to rise to more than 10% at E17.5 (**Figures 4B’’, C**). These results suggest a linear evolution of the relative endothelial surface over time (**Figure 4D**).

Angiogenesis is stimulated by VEGFA and requires the involvement of two distinct endothelial cell types: proliferating stalk cells and migrating tip cells. To mimic motility of endothelial tip cell in our model, a VEGF morphogen gradient was generated in the epithelial population by solving reaction-diffusion equations within our model. Each epithelial cell act as a VEGF source point and the gradient is obtained by imposing a fixed value for the equation solution at the boundaries (equal to zero). Tip cells were then allowed to move in the direction of this gradient by implementing a motile force acting on their vertices. Stalk cells are expected to follow the tip cell due to strong adhesion between endothelial cells (tip with stalk and stalk with stalk) and divide to fill the space generated by tip cell migration away from the original tip cell position. Here, a specific cell-cycle triggered stalk cells to divide according to their shape, *i.e.* when their elongation factor reaches a certain threshold. Evolution of the entire endothelial population depended on the VEGF-induced dynamics of endothelial tip cells. However, we also noticed that a second parameter was critical for the VEGFA-induced motility of endothelial cells: epithelial adhesion. Our simulations showed that with small values for the epithelial adhesion parameter (hence a high cohesion), and with all the other parameters kept constant, tip cells were not able to migrate inside the epithelial. Epithelial cell adhesion was thus set so that endothelial density mimics the *in vivo* situation (see **Supplementary Table 2**). Since our VM is 2-D and uses E13.5 initial conditions, it does not account for ECs moving perpendicularly to the analysed layer of tissue, nor for new ECs coming from a distant vessel. We thus integrated the formation of new endothelial branches, by the addition of newly differentiated tip and stalk cells on the periphery at a rate matching our *in vivo* quantifications. Moreover, in order to account for the fact that blood vessels within the boundaries of our simulation (defined by the epithelial mass) are connected to larger blood vessels located at a distance, and that epithelial cells are proliferating and growing outwards, we developed a set of new boundary conditions (endo-BC) for our simulation. This was essential to avoid that two growing epithelial buds of our model would fuse together and enclose the ECs. Thus, boundary vertices of every stalk cell were fixed. Furthermore, virtual boundary conditions were designed in the form of two parallel semi-infinite lines starting from the original position of the first stalk EC and elongating outward. This created new boundaries for our simulations accounting for the presence of existing blood vessels not explicitly represented. Thus, vertices belonging to epithelial cells that would position outside these endo-BC, *i.e.* between the two lines, due to epithelial growth would be constantly relocated on a new position the closest along these two lines, thereby preventing epithelial peripheral cells from two separated growing buds to merge.

**Figure 5:**
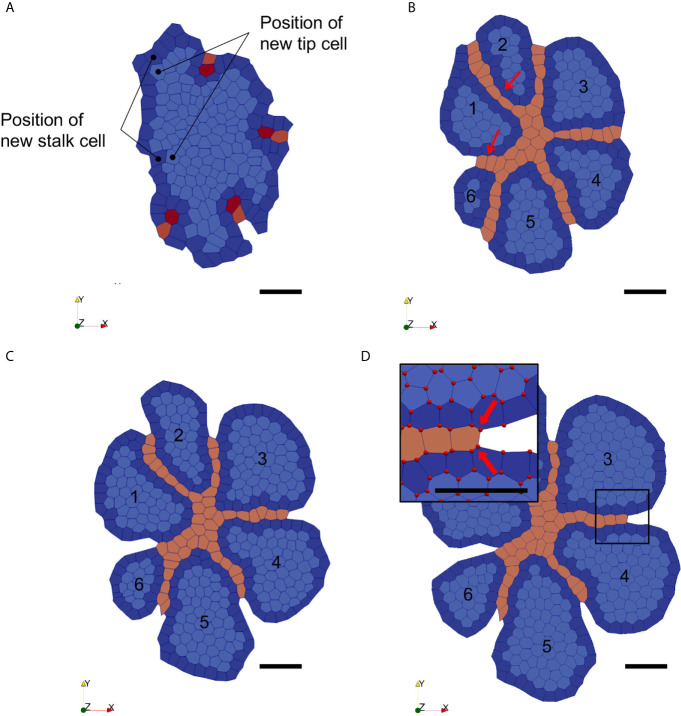
Snapshots of the VM simulation of thyroid epithelial sheet growth and endothelial invasion within the epithelial sheet. **(A)** Initial configuration of the thyroid VM (t = 0h). Epithelial cells are labelled in blue (dark at the periphery and light in the centre), endothelial tip cells are in red, and endothelial stalk cells in orange. Position of new stalk and tip cells are indicated. **(B)** Configuration at simulation time t = 10h with endothelial boundaries (Endo-BC) created for the most peripheral stalk cells. The added tip cells migrated towards the gradient, forming new vessels (red arrows). Fragmented epithelial structures are numbered (from 1 to 6). **(C)** Configuration at simulation time t = 18h. Epithelial cells continued to proliferate and grow outwardly in each bud-like structure separated by the endothelial vessels (orange). **(D)** Configuration at simulation time t = 24h. Epithelial cells continued to grow, leading to the expansion of the bud-like structures. The «virtual» endothelial vessels induced by the Endo-BC are now visible between the growing epithelial buds. The close-up view shows the vertices represented as red dots. Fixed vertices are indicated by the two large red arrows.

We then ran the simulation with our initial conditions and endo-BC, for a simulation time of 24h and a time-step of 0.001h. Cells were initialised with their specific cell cycles and with ages assigned randomly for epithelial cells. Note that, in our simulations, endothelial tip cells are considered as differentiated (hence can’t divide) and stalk cell does not divide based on cell cycle duration but on cell elongation. Initial endothelial tip and stalk cells are manually labelled based on the immunohistological images-derived initial conditions (**Figures 2A**, **5A**). Parameters for homotypic (*e.g.* epithelial to epithelial) and heterotypic (e.g. epithelial to endothelial) interactions (in eq. 2) were set to ensure a coherent and stable evolution of the system (see **Supplementary Table 2** in **Supplementary Material**), including invasion of the ECs, based on qualitative visual assessment of our simulations. In response to the VEGF gradient, endothelial tip cells thus migrated towards the centre of the epithelial mass and created “virtual vessels” which segmented the initial epithelial mass (here visualised in 2D, thus as a profile) into smaller growing masses. Virtual vessel formation is achieved by division of endothelial stalk cells and by addition of new differentiated ECs throughout the course of the simulation (**Figure 5B**, at left). We showed that the endo-BC were able to simulate the mechanical impact of (i) endothelial vessel invasion (centripetal), (ii) the presence of outward vessels, and (iii) epithelial growth (centrifuge) on thyroid mass fragmentation (**Figure 5C**). As expected, peripheral endothelial cells remained relatively fixed at their original position (**Figure 5D**, inset) while stalk cells divided at a sufficient pace to fill the space behind the migrating tip cells and ensure the formation of a coherent endothelial virtual vessel. Importantly, our simulation also showed signs of a budding-like process generated by epithelial proliferation at the periphery limited by the boundary conditions of endothelial cells (**Figure 5D**). Epithelial cells proliferated along the boundaries (accounting for the presence of endothelial cells that would connect with a distant vessel) and eventually led to the formation of smaller individual mass separated by newly formed endothelial virtual vessels.

### Cell Polarisation and Folliculogenesis

**Figure 6:**
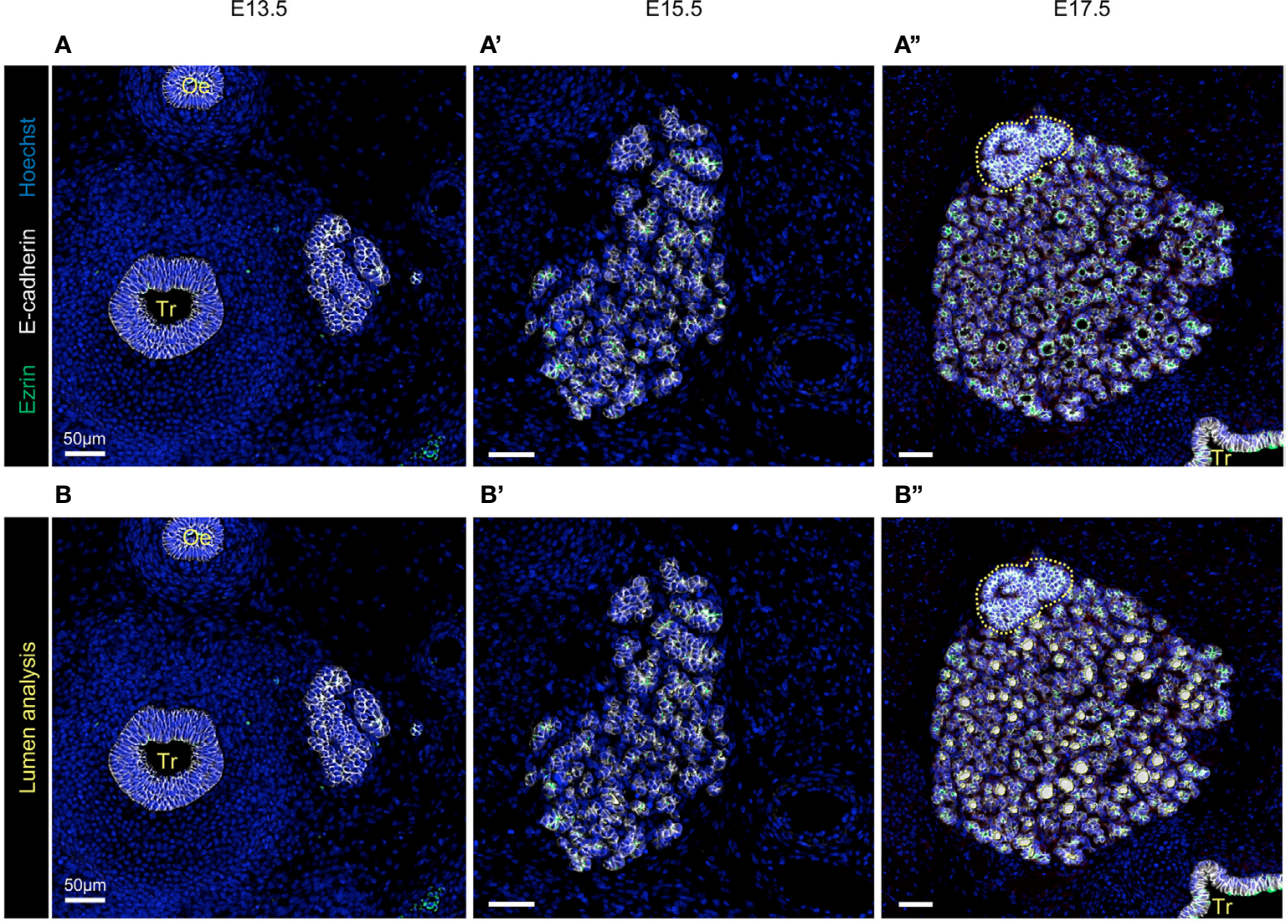
Visualisation and quantification of lumen formation in developing thyroid at three different embryonic stages. **(A–A’’)** Immunolabelling of epithelial cells in white (E-cadherin) and apical pole of epithelial cells in green (Ezrin). Nuclei are stained in blue (Hoechst). Tr, trachea; Oe, oesophagus. **(B–B’’)** Epithelial lumen within the analysed region of interest (in grey) were coloured in yellow using HALO (mostly visible in **B’** and **B’’**) and superimposed on the immunolabelling.

Folliculogenesis is the final step of thyroid embryonic development. It relies on the generation of intracellular vesicles and their fusion with the developing apical pole of epithelial cells. To integrate this process in our model we implemented the concepts of cellular polarity and lumen formation. To inform our model, we first collected quantitative data about the dynamics of lumen formation (or folliculogenesis) and growth (**Figure 6**). To visualise lumen formation and growth, we used the subapical marker ezrin (**Figures 6A–A’’**, in green). As expected, we observed a net increase in the number of epithelial cells labelled for ezrin. In fact, only trace of ezrin was detected at E13.5 (**Figure 6A**). These corresponded mainly to ezrin covering small vesicles inside the cells and not yet fused with the cell membrane. No lumen was observed between epithelial cells at E13.5. At 15.5, 43% of epithelial cells were marked by ezrin and small lumina delineated by a limited number of cells were visible (**Figure 6B’**). Finally, at E17.5 the percentage of cells labelled for ezrin rose to 80% and large lumina were evident in all the thyroid parenchyma (**Figure 6B’’**). Quantifications revealed that the absolute number of lumina increased by a factor of 3 between E15.5 and E17.5. More importantly, the mean surface of lumina increased by a factor of 13 between E15.5 and E17.5. These quantifications are consistent with previous studies (9, 10).

Folliculogenesis implies that the epithelial cells are oriented and develop apico-basal polarity. In our model, cell polarity was abstracted by the implementation of a polarity vector in every epithelial cell. This vector was characterized by a direction, accounting for the basal-to-apical polarity (i*.e.* pointing toward the apical pole) of the cell, as well as a magnitude. The magnitude precisely accounted for the so-called polarisation degree of the cell. The magnitude and direction of the polarity vector of every epithelial cell constantly varied according to their close environment.

In VMs, lumina cannot be created between cells. In fact, the topological operations (T1, T2, T3 and cell division) maintain a contiguous cell sheet with no holes between cells. Hence, we decided to represent lumina as a new computational cell type. This new cell type appears upon cell division of a mother cell with a high magnitude polarity vector (hence a high level of polarisation). The fate of the two daughter cells is determined by the position along the polarity vector: the proximal cell in contact with the basement membrane remains epithelial and replaces the mother cell with a polarity vector, while the distal daughter cell becomes a lumen cell with completely different properties (target area, adhesion, proliferation). The daughter cell that replaced the mother cell will contribute to the expansion of the newly created lumen cell. Furthermore, neighbouring epithelial cells in contact with the lumen cell will not be able to create another lumen cell, but will contribute to the expansion of the existing lumen cell. In fact, the target area of the new lumen cell will increase at a pace dependent on the polarity vectors of the surrounding epithelial cells. Hence the lumen cell will grow if the polarity vectors in the neighbouring cells gain in amplitude. Since epithelial cells in our model normally grow and divide according to their cell cycle duration, this property had to be bypassed in order to generate lumen cell when the polarity vector was gaining in amplitude and imposing cell division. Therefore, the cell cycle was modified to allow every epithelial cell to divide as soon as two conditions were met: (i) when the magnitude of its polarity vector was larger than a fixed threshold, and (ii) when the cell was old enough to divide (its age is higher than a fixed threshold).

**Figure 7:**
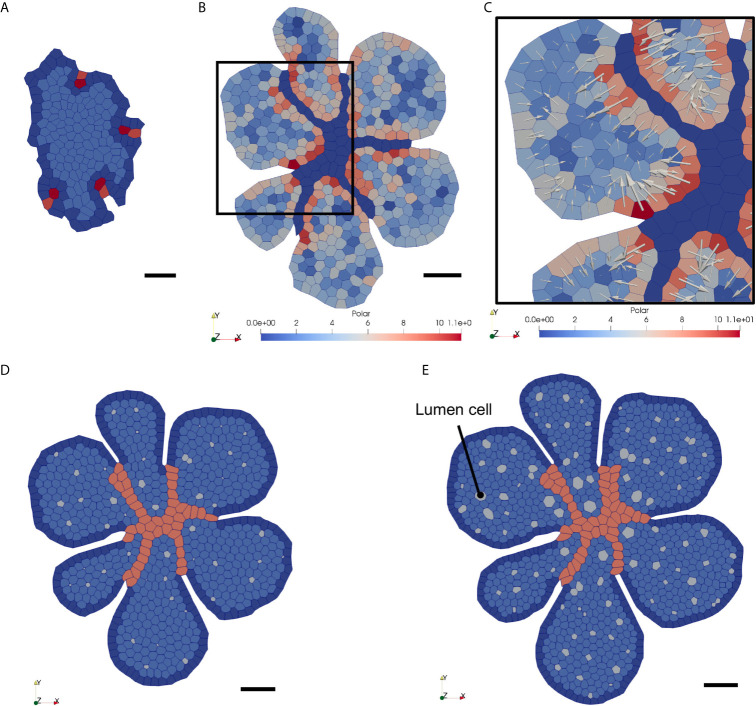
Snapshots of the VM simulation of epithelial polarisation and lumen formation in the growing thyroid epithelial sheet invaded by endothelial cells. **(A)** Initial configuration of the thyroid VM (t = 0h). Same as **Figure 5A**. No cell is polarised at that stage. **(B)** Configuration at simulation time t = 24h. Endothelial vessel network is in dark blue. Degree of epithelial cell polarisation can be visualised with the colour scale (blue, not polarised to red, polarised). **(C)** Close-up view of **(B)** with polarity vectors (white arrows) representing polarisation state for each epithelial cell. **(D)** Configuration at t = 48h. Small lumina can be observed within each of the epithelial islets. Epithelial cells keep on proliferating despite a progressive reduction in proliferation rate (see **Figure 3E**). **(E)** Configuration at t = 72h. More lumina have developed within the fragmented epithelial sheet, and lumina size continued to expand since more epithelial cells polarise. On the contrary, epithelial cell proliferation continued to decline, and expansion is thus slower.

We then ran the simulation with the same IC and endo-BC used earlier for a simulation time of 72h and with a timestep of 0.001h. As before, we manually labelled four initial endothelial tip cells and four corresponding stalk cells (**Figure 7A**). We observed that the polarisation degree of each epithelial cell, represented by its polarity vector, evolved according to its environment. Endothelial cells had a major impact on epithelial polarisation, while the impact of the boundary was slightly lower (**Figures 7B, C**, weakly-polarised epithelial cells in blue, highly-polarised epithelial cells in red). Arrows representing the polarity vectors clearly indicated that epithelial cells tended to polarise, as expected, rather orthogonally and in the opposite direction to the contact edge with their neighbouring endothelial cell, or with the epithelial mass periphery. Our model thus satisfactorily simulated the dynamic establishment of the basal to apical polarity of epithelial cells upon endothelial invasion. Furthermore, our model was able to successfully generate lumina as a result of epithelial cell polarisation, defined by the direction and magnitude of the polarity vectors. Epithelial cells started to generate small lumina after around 24h of simulation. At that point, endothelial cells had formed vessels and had segmented the initial thyroid primordium in smaller but growing individual masses. At 48h, lumina had continued to grow, due to polarised cells and new lumina also appeared from newly polarised cells (**Figure 7D**). We found that lumina that reached a significant size were developing predominantly in proximity with the infiltrating endothelium or at the periphery of the epithelial surface, which is consistent with the cell polarisation dynamics established earlier, and with our *in vivo* observations. Epithelial cell polarisation propagated across the mass triggering lumina to develop homogeneously, although with a slight delay, within the growing epithelium. After around 72h, almost 100 lumina had developed and seemed to have reached a size equilibrium with a rather circular shape (**Figure 7E**). The endothelial network did not evolve further since we did not succeed in adding more endothelial tip cells at the periphery.

## Discussion

In this work, we created a bridge between experimental science and mathematical modelling, transforming qualitative and descriptive biological information into quantitative data that can be used to drive a process-based morphogenesis model. Specifically, we used the developing thyroid as a biological system in which a mass of non-polarised epithelial cells is transformed into a multitude of polarised monolayers forming the follicles, surrounded by blood vessels. The mathematical model, developed from *in vivo* images, correctly simulated the differential growth and proliferation of central and peripheral epithelial cells, and the VEGFA-induced invasion of endothelial cells. We showed that fission of the multi-layered epithelial mass into independent pre-follicular units was controlled by endothelial cell invasion, combined with reduced epithelial cell-cell adhesion. The model could also reveal that endothelial cell abundance and proximity to epithelial cells promoted apico-basal polarisation of the latter and subsequent follicular lumen formation.

While a wide range of model types are used in computational biology, in the case of epithelial monolayers, the most commonly used framework is the two-dimensional vertex model (18). VMs are indeed often considered as highly appropriate when it comes to cell proliferation, adhesion and long-range signalling (20) and Chaste appeared as a suitable framework to develop our model. In most VMs, initial configurations are usually set as simple honeycomb-shaped cellular sheets. Here, we considered initial conditions closer to reality and directly derived initial conditions of the model from microscopic images of developing thyroid. We believe that our approach is a fast, easy but precise way to obtain realistic initial conditions for mathematical models of morphogenesis or tumour development.

Recently, Liang et al. reported and proposed a branching growth for the thyroid, similar to the pancreas or other branching organs (22). They indeed found more proliferation in subpopulations of Sox9+/Fgfr2+ cells localised at the periphery of developing thyroid lobes, as compared to more centrally located cells, thereby generating branch- or cord-like growth (22). Using EdU labelling from E13.5 to E17.5, we precisely quantified and localised cells in the S-phase of the mitotic cycle. These experimental data confirmed (i) increased proliferative potential at the periphery (60% of the peripheral cells) as compared to the centre (less than 40% of the central cells), (ii) decreased thyrocyte proliferation from E13.5 (43%) to E17.5 (10%), and (iii) allowed to compute the cell cycle duration of central (31h52) and peripheral (11h20) epithelial cells. This topological and quantitative information was instrumental for deriving key model parameters. Our observations of cell growth dynamics in the embryonic thyroid are consistent with other studies using BrdU (6, 28) and showing a peak in epithelial cells proliferation around E13.5 (32,7% of total thyroid cells) and gradually decreasing until birth to become negligible in adults (28). While we did not acquire post-natal EdU data, our computational analysis (**Figure 3D**) suggested the decrease to values close to zero in adults. Although the proliferation kinetics of adult thyroid epithelial cells have been well studied (29–31) to the best of our knowledge this is the first report providing quantitative and spatiotemporal information on the proliferation rate and pattern of embryonic thyroid epithelial cells and proposing a calculation for cell cycle duration of peripheral and central cell populations. Our observations that the majority of EdU^+^ cells are located at the periphery of the thyroid primordium, combined with those of Fagman (6) and Liang (22), suggest that thyroid epithelial growth could rely on the existence of proliferating epithelial tip cell subpopulations as described in the pancreas (23). One possible explanation for the appearance of these peripheral epithelial subpopulations is the existence of mesenchymal niches as recently demonstrated in the pancreas (32).

Our model correctly simulated the formation of an endothelial network within the epithelial mass (here modelled as a sheet), in response to a VEGFA-dependent gradient. Thyroid tissue observation reveals that endothelial cells are found at the periphery of the lobes at E13.5 and then everywhere in the thyroid parenchyma at E17.5 (6, 9). Recruitment and expansion of endothelial cells during thyroid development depends on the expression of VEGFA by all the thyrocyte progenitors (9). We thus imposed the expression of VEGFA to the epithelial cell type forming our initial condition. This resulted in the formation of a centro-peripheral gradient of VEGFA that induced migration and invasion of endothelial cells towards the centre of the epithelial sheet. Peripheral to central ECs invasion, concomitant with outward expansion of the epithelial sheet, allowed fragmentation or fission of the initial sheet in smaller growing epithelial islets. Transformation of the initial epithelial sheet into smaller islets was only possible when the issue of endothelial boundaries was solved by imposing “no man’s land” regions behind the most peripheral ECs. These endo-BC were essential to simulate the connection with distant peripheral vessels. Indeed, these connections are not visible in 2D histological sections and were thus absent from the initial condition of our vertex model. We faced two other problems with ECs in our 2D VM that we could not solve and that led to under-representation of ECs. First, ECs coming from the third dimension, *i.e.* from above or below the analysed plane or epithelial sheet, could not be modelled. Secondly, due to stability issues, appearance of new tip cells was possible during the first 24h of our simulation but not beyond. This was a deception because we expect that new tip cells should appear from the newly formed virtual vessels to invade the newly formed epithelial islets. Indeed, the VEGF gradient is evolving with modelling time and upon fragmentation of the initial sheet and the appearance of new epithelial islets, secondary VEGFA gradients should develop in every islet, thereby redirecting ECs migration. Moreover, anchoring specific vertices (*i.e.* boundary vertices belonging to the most external stalk cells) adds another level of complexity to the model. However, without anchoring these vertices, the ECs would simply behave like any other epithelial cells, even with different adhesion parameter values, and would always relax to a spherical configuration with no fragmentation of the initial sheet. Yet by anchoring these vertices, we had to ensure that the epithelial cells belonging to different islets would not fuse and eventually lead the simulation to similar results hence the development of specific endo-BC. However, junctional rearrangements are still performed on endothelial cells and can lead to the motion of vertices that we would expect not to move, typically for peripheral stalk cells. This can lead to very small, if not too small edges, that our simulations can no longer handle. Working on these aspects of the model is a priority.

Moreover, while VMs are generally accepted as a powerful tool to simulate epithelial cell sheets, endothelial cells exhibit notable differences in their shape and proliferative behaviour. Various mathematical and computational models tackling the issue of angiogenesis exist, ranging from complex continuous 3D models such as “Microvessel Chaste” (33) to simpler cell-base models (34–36). In our thyroid model, we described endothelial and epithelial cells in the same framework in order to study the various impact of the growing endothelial network on epithelial cell polarisation and organisation in thyroid follicles. We showed that ECs motility, proliferation and adhesion were acceptably simulated in our model, despite some inaccuracies (notably regarding ECs size and density). However, as already discussed, stability issues in our model did not allow for a continuous development of the endothelial network, therefore preventing the differentiation of new tip cells throughout the course of simulation.

It should also be noted that besides ECs expansion and invasion, epithelial cell adhesion appeared as a second critical parameter for the fragmentation, or fission, of the thyroid lobes. Initial simulation indeed revealed that ECs invasion towards the centre of the sheet was only possible with low adhesion between epithelial cells. This observation is particularly interesting since at the stage analysed in our study, besides the classical epithelial (E) cadherin, another cadherin molecule, R-cadherin (Renal-specific cadherin), now called cadherin-16, has been reported to be induced in the thyroid epithelium and maintained in the adult thyroid (37). Furthermore, a role for cadherin-16 was proposed in the developing thyroid when it was observed that E-cadherin (Cdh1) knockout had normal follicles and only caused mild defects to the organ (38). Cadherin-16 has also been identified as a downstream target of the transcription factor Pax8, expressed in the thyroid and kidney (39). More recently, using thyroid cells in culture, Pax8 was found to control apico–basal follicular polarisation and follicle formation through Cdh16 (40). In addition, silencing of Cdh16 expression led to the formation of defective follicular structures characterised by very low laminin expression at the follicle–matrix interface (40). Interestingly, in Smad1/5 double knockout thyroid that present defective thyrocyte polarisation and follicle formation (10), expression of Cdh16 was also dramatically reduced at E14.5 (Villacorte and Pierreux, unpublished). To compare the abundance of these two cadherins, we measured the absolute number of mRNA molecules for E-cadherin (Cdh1) and R-cadherin (Cdh16) in developing thyroid (Spourquet and Pierreux, unpublished). Although we measured 16X more *Cdh1* as compared to *Cdh16* mRNA molecules at E13.5, we found 1.2X more *Cdh16* than *Cdh1* molecules at E17.5. In other words, and if compared to epithelial cell abundance, while at E13.5 each epithelial cell contained 50 *Cdh1* and 3 *Cdh16* mRNA molecules, at E17.5 each epithelial cell contained 9 *Cdh1* and 11 *Cdh16* mRNA molecules. Thus, less Cdh molecules per cell and a different ratio of these two cadherins. These data on Cdh16 combined with our observation that epithelial cell-cell adhesion should be reduced to allow ECs invasion suggests that Cdh16 expression at the time of folliculogenesis could reduce epithelial cell-cell adhesion by interfering with Cdh1.

Our model can be used to test the importance of biological parameters. Knowing the *in vivo* effect of VEGFA KO on thyroid folliculogenesis (9), our model was validated by suppressing the VEGFA morphogen production (hence diffusion) *in silico* (**Supplementary Figure 2**). In the absence of VEGFA production in the epithelial sheet, endothelial tip cells do not migrate and stay relatively immobile at their initial position. Epithelial cells proliferated inducing the growth of the epithelial sheet. New endothelial cells were added, but migration was not observed, and no endothelial network was formed. In addition, the endothelial density after 48h was significantly lower than in the presence of a VEGFA gradient. The few endothelial cells present had a polarisation effect on surrounding epithelial cell, as well as the periphery of the epithelial sheet. This polarisation effect was nevertheless too low to generate large and numerous lumina. The similarity between this simulation and the phenotype of the VEGFA KO indicates that the various processes and parameters are correctly implemented.

Our model also simulated apico-basal polarisation of epithelial cells and subsequent follicular lumen formation. We developed and used an epithelial polarisation model by assigning a polarity vector to every epithelial cell. This allowed an explicit definition of the polarisation degree of epithelial cell required before generating a lumen. Various models of cell polarisation have already been developed (41). However, these models do not integrate the mechanical interactions of endothelial vessels with the epithelium. Here, the implementation of our apico-basal polarity model allowed to consider different cell populations and their effect on each other within the same framework. We were in fact able to simulate the polarisation of epithelial cells depending on their proximity to ECs, lumen and other epithelial cells.

Our model, as all VMs, represent cells as polygons. Vertices are linked together by edges to form cells, generating a coherent population. However, the very definition of VMs prevents the existence of stable “voids” in the simulation. In order to get around these model limitations, lumina were defined here as a distinctive cell type. The easiest way to generate new cells in VMs is simply by cell division. Adding vertices and cells is technically possible but difficult when it comes to generating new cells inside the epithelial cell population. Lumina were therefore generated by the division of an epithelial mother cell in two daughter cells. One daughter cell remained epithelial and the other one became a lumen. We developed our polarisation model so that the lumen cell would develop at the “apical edge” of the mother cell (*i.e.* at the extremity of the mother cell polarity vector). This process actually mimics the transport of intracellular vesicles loaded with apical material observed *in vivo* (9, 10, 42). The area occupied by the newly created lumen cell was instantly set to lower value; its size only increased upon concerted orientation of polarity vector from neighbouring cell. Lumen is known to have a spherical form (as observed in **Figure 1C**). It is obviously not the case in our simulation since lumen is represented by particular polygonal cell, defined by vertices and edges. A circular form will therefore never be achieved, but each lumen will grow in size as a function of the number and polarity of epithelial cells surrounding it. We thus believe our model satisfactorily simulate *in vivo* lumen formation. Moreover, the number and localisation of these lumina were coherent with our observations and quantitative analysis.

Models should not be judged by their complexity or level of details, but based on what we learned from them. Here, the definition and implementation of numerous modules, accounting for various processes involved in the morphogenesis of the embryonic thyroid, altogether gave encouraging results. By using a model, we advanced our understanding of the thyroid gland morphogenesis. We quantitatively characterised the proliferation of epithelial cells and extracted initial conditions from *in vivo* images to be more coherent with the observed biology. Our model also allowed us to illustrate the crucial role of changes in cell-cell adhesion in the invasion of endothelial cells and the fission of the initial epithelial mass and of epithelial cell polarisation triggering follicle formation. Such a study would have been much more difficult to achieve by following only a classical *in vivo*/*in vitro* experimental approach, especially considering the very high complexity involved in the inference of cell adhesion *in vitro*. We firmly believe that *in silico* approaches will keep on developing and that our results paved the way for more advanced studies in the field of mathematical biology.

## Data Availability Statement

The original contributions presented in the study are included in the article/**Supplementary Material**. Further inquiries can be directed to the corresponding author.

## Ethics Statement

The animal study was reviewed and approved by Commission d’Ethique pour l’Expérimentation Animale Secteur des Sciences de la Santé Université catholique de Louvain.

## Author Contributions

LG conceived and performed all the image and computational analysis, prepared the modelling figures and wrote the manuscript. CS and PL collected the biological material and performed the immunolabelling experiments. CS prepared the biological figures. MB was involved in the proliferation analysis and LL in the polarisation and lumen formation. AF developed and provided important inputs with Chaste. EH and CP supervised the project and wrote the manuscript. All authors contributed to the article and approved the submitted version.

## Funding

This work was supported by a grant from the Université catholique de Louvain (Actions de Recherche concertées, ARC 15/20-065 to EH and CP). The core facility for Imaging Cells and Tissues was financed by National Lottery, Région bruxelloise, Région wallonne, Université catholique de Louvain, FNRS and de Duve Institute.

## Conflict of Interest

The authors declare that the research was conducted in the absence of any commercial or financial relationships that could be construed as a potential conflict of interest.

## Footnotes

1
https://indicalab.com/halo/

2
https://mathworks.com/products/matlab.html

3
https://chaste.cs.ox.ac.uk

4
www.paraview.org
