# Supplementary material for: Modelling of Epithelial Growth, Fission and Lumen Formation During Embryonic Thyroid Development: A Combination of Computational and Experimental Approaches
Source: Front Endocrinol (Lausanne). 2021 Jun 7;12:655862. doi: 10.3389/fendo.2021.655862 (PMC8216395; doi:10.3389/fendo.2021.655862)
Supplement: Supplementary file 1 [file DataSheet_1.pdf]

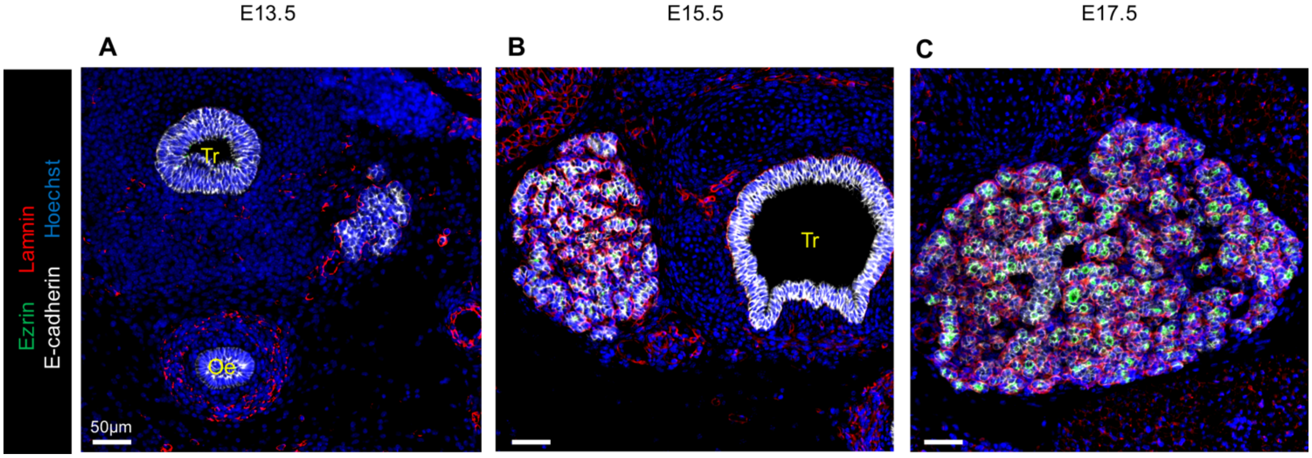

Supplementary Figure 1. Visualisation of apico-basal polarization at three different embryonic stages. Immunolabelling of epithelial cells in white (E-cadherin), basement membrane in red (Laminin), and of the apical pole of epithelial cells in green (Ezrin). Nuclei are stained in blue (Hoechst). Tr, trachea; Oe, oesophagus. (A) At E13.5, non-polarised epithelial cells are organised in a multi-layered mass surrounded by the basement membrane (here, the right thyroid lobe is shown). Magnification revealed that only a few cells present weak intracellular Ezrin labelling, but no intercellular lumen. (B) At E15.5, the thyroid lobe has greatly increased in size and is progressively losing its multi-layered aspect. Magnification revealed that the thyroid is now fragmented in cords or small islets of epithelial cells surrounded by a basement membrane (red). Ezrin+ structures are now easily visible and represent small interepithelial lumina. (C) At E17.5, the thyroid has reached an almost mature configuration, with epithelial cells organised in monolayers delineating a central lumen (follicular lumen in green) and surrounded by a basement membrane (red).
