# Supplementary material for: Modelling of Epithelial Growth, Fission and Lumen Formation During Embryonic Thyroid Development: A Combination of Computational and Experimental Approaches
Source: Front Endocrinol (Lausanne). 2021 Jun 7;12:655862. doi: 10.3389/fendo.2021.655862 (PMC8216395; doi:10.3389/fendo.2021.655862)
Supplement: Supplementary file 2 [file DataSheet_2.pdf]

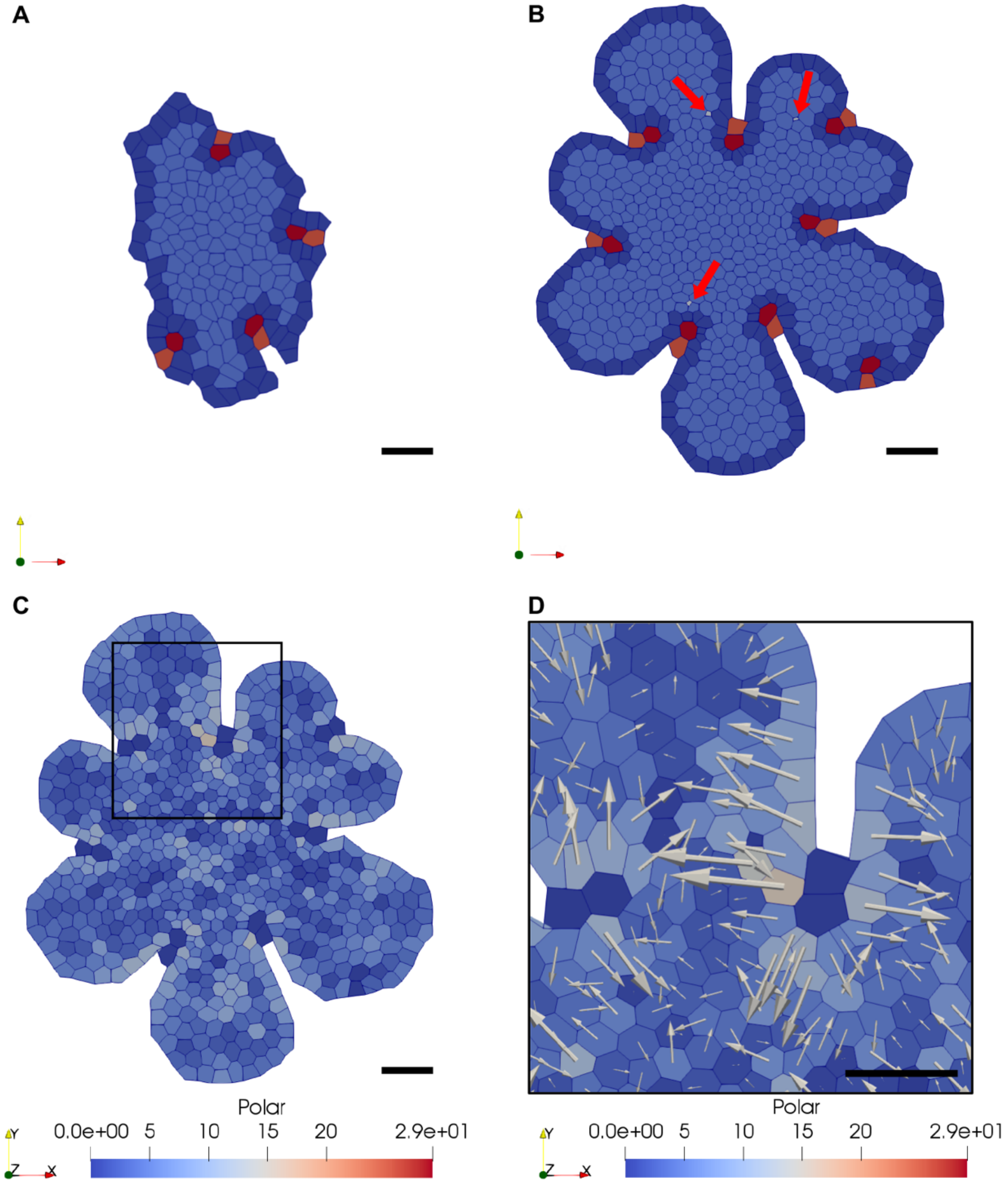

Supplementary Figure 2. Snapshots of the VM simulation of thyroid epithelial sheet growth and endothelial invasion in the absence of morphogen diffusion. (A) Configuration at initial time. Endothelial tip cells are labelled in red, endothelial stalk cells in dark orange. Each tip cell is directly followed by a stalk cell. (B) Configuration at simulation time t = 48h with no morphogen diffusion. Stalk and tip cells remained at their initial position and did not migrate. New endothelial cells were added throughout the simulation. Small lumina (indicated by red arrows) can be observed due to the polarisation of epithelial cells in close proximity with existing endothelial cells and/or the periphery of the cell population. (C) Configuration at simulation time t = 48h with no morphogen diffusion. Cells are coloured according to their polarisation level. (D) Close-up view of (C). Cell polarity is represented by an arrow (polarity vector) for each epithelial cell. Arrows are scaled according to the magnitude of the polarity vector.
