# Supplementary material for: Modelling of Epithelial Growth, Fission and Lumen Formation During Embryonic Thyroid Development: A Combination of Computational and Experimental Approaches
Source: Front Endocrinol (Lausanne). 2021 Jun 7;12:655862. doi: 10.3389/fendo.2021.655862 (PMC8216395; doi:10.3389/fendo.2021.655862)
Supplement: Supplementary file 3 [file DataSheet_3.pdf]

Table 1. Antibodies and dilution

Antibody	Supplier	Reference	Species	Dilution
E-cadherin	BD Biosciences	610182	Mouse IgG2a	1/500
Endomucin	Santa Cruz	sc-65495	Rat	1/1000
Ezrin	Thermo Scientific	MS-661-P1	Mouse IgG1	1/400
Laminin	Sigma	L9393	Rabbit	1/100

Table 2. Model parameters values

Parameter	Description	Value	Dimensions
K	Deformation energy parameter	55.0	[Force.Lenght^{-1}]
$\Lambda_{epi-epi}$	Epithelial cell-cell adhesion energy parameter	5.0	[Force]
$\Lambda_{endo-endo}$	Endothelial cell-cell adhesion energy parameter	1.0	[Force]
$\Lambda_{lumen-lumen}$	Lumen cell-cell adhesion energy parameter	5.0	[Force]
$\Lambda_{epi-endo}$	Epi-endo cell adhesion parameter	5.0	[Force]
$\Lambda_{epi-lumen}$	Epi-lumen cell adhesion parameter	6.0	[Force]
Γ	Contractility parameter	1.0	[Force.Lenght^{-1}]
ρ_{endo}	Polarisation impact rate of endothelial cells	0.12	[Time^{-1}]
ρ_{bnd}	Polarisation impact rate of the periphery	0.12	[Time^{-1}]
ρ_{lumen}	Polarisation impact rate of lumen cells	-0.015	[Time^{-1}]
ε	Polarisation impact rate of epithelial cells	-0.04	[Time^{-1}]
